# Escaping the Environmental Crises: Online Escape Rooms for Evaluating Student Data Analysis Skills

**DOI:** 10.1021/acs.jchemed.3c00339

**Published:** 2023-10-23

**Authors:** Angelica R. Cash, Julia R. Penick, Celia F. Todd, Monica C. So

**Affiliations:** † California State University, Chico, Chico, California 95929-0210, United States; ‡ University of California, Santa Cruz, Santa Cruz, California 95064, United States

**Keywords:** Undergraduate/General, Distance Learning/Self Instruction, Internet/Web-Based Learning, Laboratory Instruction, Collaborative/Cooperative Learning, Humor/Puzzles/Games

## Abstract

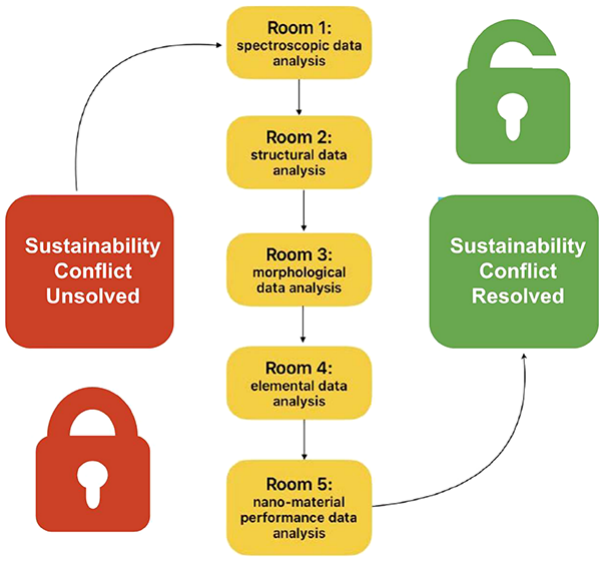

Summative lab assessments probe student mastery over concepts, but conventional ones often result in decreased student engagement and confidence. If conventional summative lab assessments are replaced by accessible gamified evaluations, such as online escape rooms, this leads to improved student engagement and confidence. In this work, we adapted two sustainability themed online escape room activities to increase student engagement and confidence in data analyses in Integrated Chemistry I (CHEM 381) over three semesters at CSU, Chico. Over 89.7% of students earned full credit. Further, 80.0% of the written comments included positive feedback. After the online escape room assessments, 60.0% of the students rated their confidence as “high” or “very high” in all categories assessed, compared to 25.6% before the experience. Students found that the online escape room assessments were more engaging than the traditional assessment and increased their confidence as they worked toward solving two sustainability crises and competed for the quickest time to complete the escape rooms.

## Introduction

Throughout academia, conventional assessments (e.g., essays, midterms, finals) are often used to measure student learning. However, gamified assessments may increase student engagement and confidence.^1,2^ Instructors have recently introduced escape rooms into the classroom to encourage students to work through puzzles and solve a specific goal within a given timeframe, typically under an hour. Several works^3−6^ found that escape rooms promote social interaction and communication.^7,8^ Further, students reported learning gains,^7^ increased motivation,^9,10^ and a more positive learning experience^11^ through a gamified escape room.^5,14,15^

During the pandemic, virtual activities such as online escape rooms^23−27^ and augmented reality activities^29,30^ became necessary to enhance learning and retention. Previous chemistry instructors adopted various platforms, such as Google Forms^12^ and WhatsApp,^13,21^ to conduct virtual escape room activities and evaluate student understanding in different chemistry subdisciplines. To address misconceptions in general chemistry while improving learning motivation and increasing student engagement, Cai designed a Harry Potter themed online escape room.^22^ Similarly, Vergne and co-workers evaluated student understanding of fundamental organic chemistry in a chocolate factory themed digital escape room.^20^ To help pharmacy students learn the complex topic of stereochemistry, Abdul Rahim found that the students became more engaged when participating in online escape rooms to solve puzzles rather than listening to a traditional lecture.^16^ Lopez-Pernas et al. noted that while in-person and remote educational escape rooms improved students’ knowledge in computer programming, in-person escape room participation yielded slightly higher postactivity test scores compared to the remote versions.^24^ Interestingly, unlike the previous studies, another involved undergraduates designing their own digital escape rooms with peers in an introductory ecology course, leading to increased engagement.^17^

However, this work differs from previous ones in several ways. First, these escape rooms center on sustainability themes, such as solar energy conversion and water decontamination. The students were tasked with finding experimental methods to build the most efficient solar cell^18,19^ along with water filtration materials^20^ to prevent environmental crises. Second, the students require intradisciplinary knowledge to solve the puzzles in the escape rooms created for this work. They relied on combining prior knowledge in general, organic, inorganic, physical, analytical, and materials chemistry to be successful in data analyses. Specifically, the students needed to interpret various data types, such as X-ray diffraction (XRD), scanning electron microscopy (SEM), ultraviolet–visible (UV–vis) spectroscopy, Fourier transform infrared (FT-IR) spectroscopy, steady-state fluorescence spectroscopy, adsorption data, and electrical characterization. We previously evaluated individual student’s skills acquisition through questions (Supporting Information) in the final paper written by each student.^18,20^ Third, the online escape rooms created in this work target data analyses in the upper division integrated undergraduate chemistry laboratory (CHEM 381) at CSU, Chico. Since in-person learning opportunities were minimal during the pandemic, gaining valuable exposure to different data analyses was essential to chemistry students’ educational and professional development.

In this work, we designed two accessible online escape room activities to increase student engagement and confidence in data analyses in the CHEM 381 course at CSU, Chico. These online escape rooms were administered utilizing user-friendly Google Forms and moderated in an online-learning environment using Zoom breakout rooms. To increase accessibility to more students, these escape rooms were executed remotely without any use of paper. To increase engagement, the puzzles involve answering questions to solve a sustainability themed crisis in under an hour. To evaluate changes in student confidence, students self-rated their confidence levels in each form of data analysis before and after the online activity. The questions in the online escape rooms are all based on laboratory projects^18,20^ during the CHEM 381 course. These questions are also modular in rigor, question type, and concepts tested. Consequently, the online escape rooms can be adapted for high school and all levels of undergraduate chemistry classes.

## Methodology

### Virtual Escape Room

For a semester-long course, students virtually participated in two laboratory projects^18,20^ in an upper-level integrated chemistry laboratory course (CHEM 381) at CSU, Chico. CHEM 381 is designed for chemistry and biochemistry majors to apply concepts and techniques from their inorganic, physical, and analytical chemistry courses into research-based projects. For each laboratory project, students spent 7–8 weeks familiarizing themselves with analyzing structural (X-ray diffraction, XRD), morphological (scanning electron microscopy, SEM), spectroscopic (UV–vis absorption spectroscopy, UV–vis), elemental composition (energy dispersive X-ray spectroscopy, EDXS), and performance metric data (efficiency, η; adsorption capacity, *q*_t_) of the nanomaterials). At the conclusion of each project, a virtual escape room activity was used to assess the student’s proficiency at interpreting data of each type (Table S1).

### Designing the Digital Escape Room

Designing the digital escape room includes the following:

- Goals:
  (a) Increase accessibility of assessments to more students.
  (b) Increase student engagement through the activity.
  (c) Increase students’ confidence levels in data analyses relevant to this course.

- Sustainability Themes:
  (a) Lab 1. Renewable Energy Crisis
  (b) Lab 2. Water Decontamination Crisis
- Player Control: Players cannot advance to the next puzzle until they enter the correct numerical or textual answer to solve the current puzzle they are in.
- Group Size: Three students per group maximizes the amount of work each player is required to complete during the escape room.
- Time Constraint: Triads of students will have 60 min to complete the virtual escape room puzzles. Students who do not complete the puzzles within the hour only earn the percentage of total points based on the number of puzzles completed.
- Difficulty: Players must have knowledge of undergraduate-level general, inorganic, organic, physical, analytical, and materials chemistry. Players in this study were composed of majors-level biochemistry and chemistry students.
- Feedback: Feedback was collected immediately after the students’ game experience through surveys and verbal responses.

### Implementation of Online Escape Room

In total, 90 students virtually participated in this study over three semesters. Specifically, 14 triads of students completed the energy crisis escape room, while 16 triads of students completed the water decontamination escape room. Once students were randomly divided into Zoom breakout rooms to form these triads, a predesigned Google Form escape room was released to each student simultaneously (Figures S4 and S6). Each room consisted of a data type which emphasized understanding of structure–property relationships (Supporting Information). The data that students analyzed included the following: room 1, relating absorption spectra to structure of materials; room 2, confirming crystal structure via XRD patterns; room 3, relating morphology (size, density, shape) of nanomaterials with electron microscopy images; room 4, correlating elemental composition to spectroscopy signals; room 5, quantifying performance of each nanomaterial. As each section of the Google Form was completed with correct answers, the groups moved forward to the next section or “room” (Figure S8). Incorrect answers prevented groups from advancing to the next section or “room” (Figure S9). Full points were earned by triads of students by completing the five rooms of the escape room within 60 min; if students completed one room, they earned 20% of the total points. Thus, if all five rooms were successfully completed, students earned 100% of the points. Groups competed for completing the escape rooms in the shortest time, which resulted in additional excitement and incentive for the students. Times were calculated from the start time to the finish time at which the groups submitted their Google Form, i.e., successfully completed the assigned escape room activities.

### Modifications

The escape room was adapted to apply to the second experiment performed during the semester. Students had synthesized metal–organic frameworks (MOFs) for use in water decontamination of dyes and oils.^28,29^ The second escape room consisted of a water decontamination crisis that needed to be solved (Figure S6). First, characterization of the MOFs needed to be completed to proceed through the escape room, including analysis using powder X-ray diffraction, scanning electron microscopy, energy dispersive X-ray spectroscopy, and Fourier transform infrared data. To complete the escape room, the students were required to determine the adsorption capacity of dyes compared to oils. While given the same amount of time and same size groups, the escape room questions were easily changed to meet the needs of the new project assessment.

## Results and Discussion

**Figure 1:**
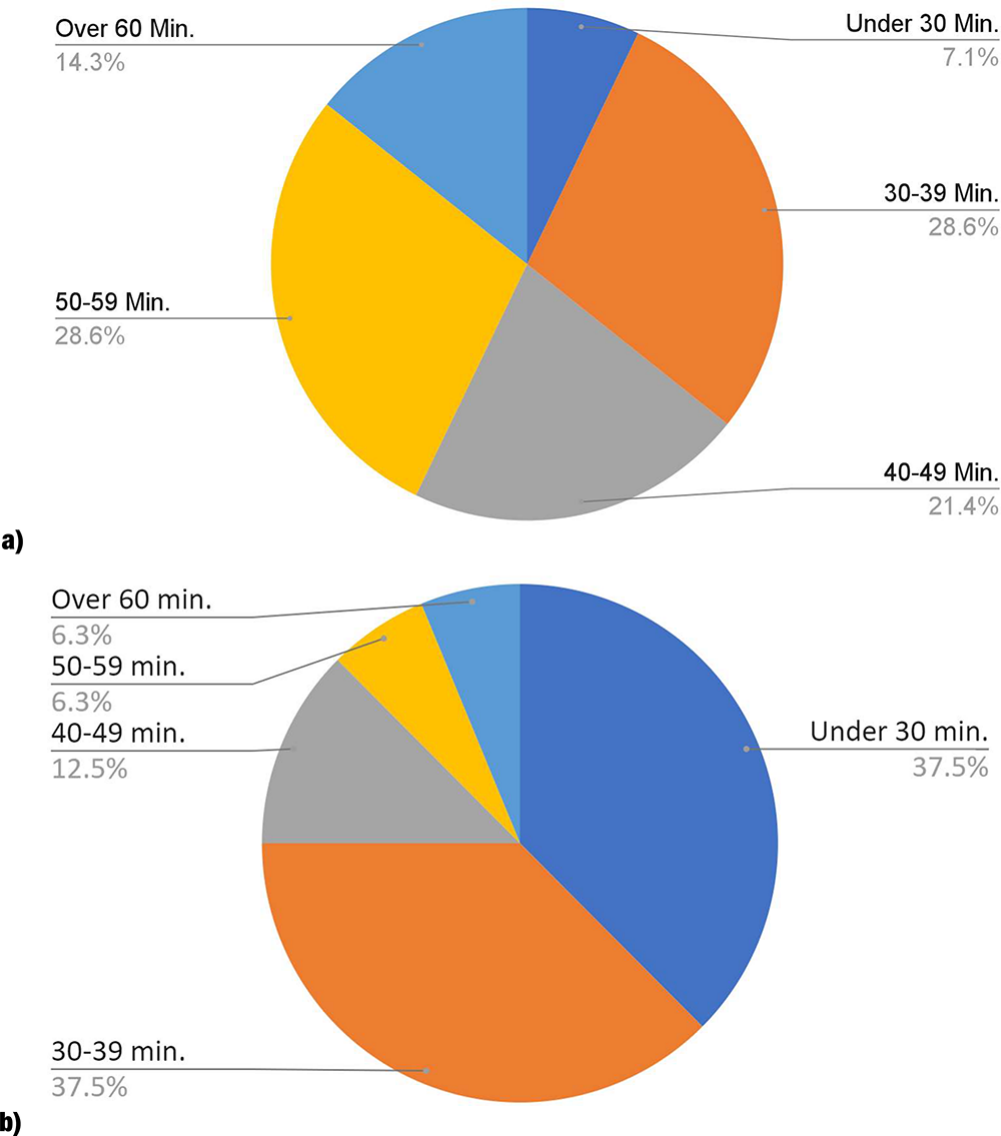
Group completion times of (a) energy crisis escape room and (b) water decontamination crisis escape room.

Of the groups, 85.7% completed the energy crisis escape room in under 1 h, and 93.7% of groups completed the water decontamination crisis escape room within the 1 h time limit (Figure 1). Students earned credit based on the completion; therefore, an average of 89.7% of the groups that participated received full credit. The completion times can be indicative of the difficulty of the escape room. The above results show that the escape rooms were made with adequate difficulty for the students of the upper division integrated chemistry laboratory. If needed, the amount of time required to complete the escape room could easily be adjusted to meet the level of the demographic by changing the questions within the Google Form. For example, a fill in the blank could be changed to multiple choice.

Group completion times decreased after prior exposure to the escape rooms. Approximately 35.7% of students completed the energy crisis escape room within 40 min, compared to 75.0% of students in the water decontamination escape room. After exposure to the first virtual escape room activity, the students were more prepared for the expectations of the second virtual escape room activity. They gained familiarity with using the Google Form application as well as the types of questions they would encounter. Students were also better able to build the skills of data analysis throughout the semester. Thus, they were more experienced with the analyses that they solved within the escape rooms.

It was noted that students had commented that solving an efficiency equation had taken them a long time to complete. This could have been a factor in the length of time that the students had taken to complete the energy crisis escape room as there was not an energy efficiency equation within the water decontamination escape room.

**Figure 2:**
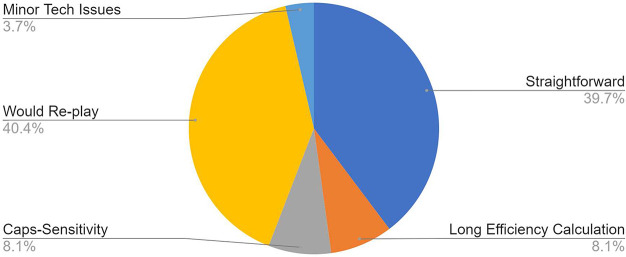
Student feedback on escape rooms.

To monitor how students felt about the escape rooms, a short discussion was held once each was finished (Figure 2). The discussion consisted of short yes/no questions, but it also allowed students to give feedback for how the escape rooms could be improved in the future. Overall, 80.0% of the comments included positive feedback, while 8.10% of students experienced a specific challenge of spending most of their time solving a “long efficiency equation”. The remainder of the feedback reflected the technical issues experienced, such as caps-sensitivity issues for answers entered into each field of the Google Form.

**Figure 3:**
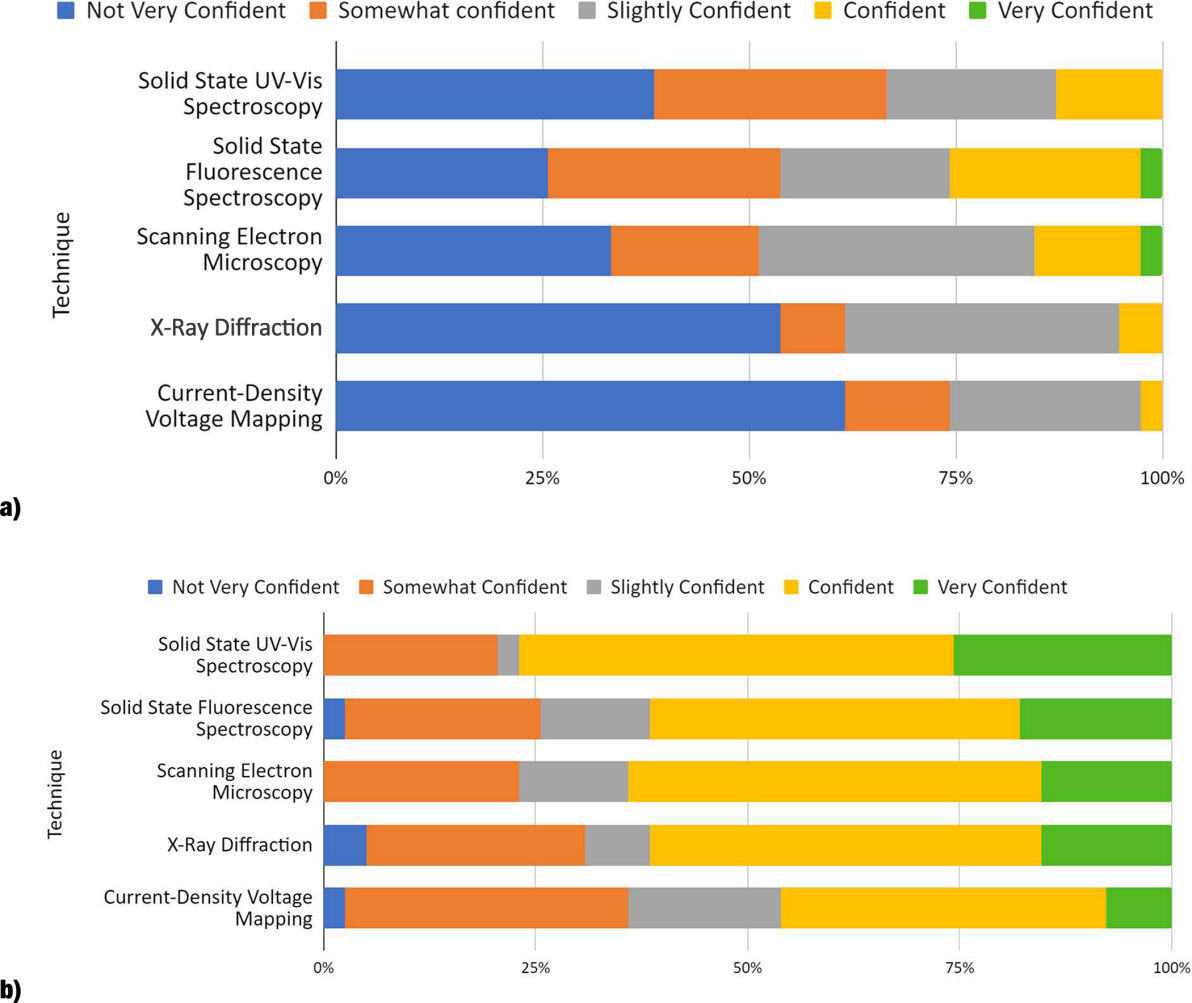
Student confidence levels (a) before and (b) after energy crisis escape room.

In order to assess student confidence in specific lab techniques used in this course, students completed the same survey comparing self-assessed confidence levels before and after the escape room was completed (Figure S7). The students did not receive practice escape room questions and so did not know what kind of questions to expect before the activity. These are captured as more blue colors, denoting low confidence levels in Figure 3a. After working through and completing the questions in the online escape rooms, the students’ confidence levels increased, as shown by the appearance of more green colors (i.e., higher confidence levels) in Figure 3b.

Compared to the 25.6% of students who were confident or very confident in all lab techniques assessed in the energy crisis escape room, 60.0% of the students rated their confidence this way after the escape room in all techniques except current density–voltage mapping. This is consistent with findings in Figure 2, since 8.1% of students indicated that the efficiency calculation took a long time to complete. Factors that may have an impact on the confidence levels of students could be previous analytical experience from other chemistry courses. For example, UV–vis spectroscopy is used in lower division chemistry courses, as well as organic chemistry courses and quantitative analysis courses, as opposed to X-ray diffraction and current density–voltage mapping, to which they have not been exposed prior to the CHEM 381 course.

## Conclusion

In summary, online escape rooms using Google Forms are an accessible method for assessing students’ proficiency of data analysis in a majors-level chemistry laboratory at CSU, Chico. During the escape room, students were engaged, since an average of 89.7% completed the online activities in under 60 min, and 80.0% of the written comments about the activity were positive. Since many of the groups completed the entire escape rooms in under 60 min, the difficulty of the escape rooms was adequate for our participants at CSU, Chico. The students wrote that they welcomed participating in the online escape rooms again and found that the escape rooms were more fun than the traditional exam. It helped that students worked toward solving sustainability themed crises and competed for the shortest time to complete the escape rooms. After the activity, 60.0% of the students rated their confidence as “high” or “very high” in all categories assessed, compared to 25.6% before the experience.

These online escape rooms benefit from being highly adaptable. First, since students are returning to in-person classes, the online escape rooms can be adapted in a classroom setting. Students can be split into breakout rooms on their devices through Zoom and work together as a group to complete the escape room while in the classroom. This can increase student interaction and lead to increased student performance. Furthermore, the difficulty and time of completion may be modified to meet the demographic of the targeted students of other future work. For example, escape rooms using Google Forms can also be modified to meet the abilities of lower division courses or secondary school students. These escape rooms evaluated the student’s ability to interpret data, but it could also be applied to other fields of chemistry. As we have adjusted from an energy crisis themed escape room to a water decontamination themed escape room, we changed the types of analyses that students completed to reflect the knowledge they had gained from the water decontamination data analysis.

## Limitations

The activity could benefit from more control variables planned during the experimental setup. Future implementation of Escapp,^30^ a web platform that allows teachers to conduct remote and face-to-face escape rooms, would allow for a deeper quantitative analysis. This platform allows for teachers to obtain various learning analytics such as progress graphs that show the puzzles each group solves and the moment in which each one was completed. This would be useful information to understand which topics are more challenging than others. Escapp also provides a hint chart which shows the number of hints provided to each team for each puzzle. Monitoring the hints would allow teachers to understand which specific topics students struggle with the most.

Another limitation to the study included technological challenges with Google Forms. Some groups experienced difficulties with caps-sensitivity issues and using the incorrect number of decimal places, which slowed their progress. This could be improved in the future by changing the Google Form settings to be less sensitive prior to the escape room being conducted. Clarifying the number of decimal points before completing the escape room would have saved students time with numerical answers.

Finally, this study can be improved by evaluating the effect of the online escape room activities on teamwork skills and stress levels on students. By administering surveys pre- and post-activity to the students, researchers can further investigate essential teamwork skills such as active listening, cooperation, conflict resolution, coordination, creativity, feedback, and problem solving. To assess stress levels on students, a Likert scale questionnaire ranging from 1 “no stress” to 5 “extreme stress” before and after the activity can be implemented.

## Supporting Information Available

Example brief descriptions; customization instructions; notes for instructors including descriptions of the content for the virtual escape rooms (PDF)

## References

[ref1] Veldkamp A.; van de Grint L.; Knippels M.-C. P. J.; van Joolingen W. R. Escape Education: A Systematic Review on Escape Rooms in Education. Educ. Res. Rev. 2020, 31, 100364 10.1016/j.edurev.2020.100364.

[ref2] Gómez-Urquiza J. L.; Gómez-Salgado J.; Albendín-García L.; Correa-Rodríguez M.; González-Jiménez E.; Cañadas-De la Fuente G. A. The Impact on Nursing Students’ Opinions and Motivation of Using a “Nursing Escape Room” as a Teaching Game: A Descriptive Study. Nurse Educ. Today 2019, 72, 73–76. 10.1016/j.nedt.2018.10.018. 30453202

[ref3] Fotaris P.; Mastoras T. Escape Rooms for Learning: A Systematic Review. In Proceedings of the 12th European Conference on Game Based Learning; ACPI: 2019; p 30. 10.34190/GBL.19.179.

[ref4] Sánchez-Martín J.; Corrales-Serrano M.; Luque-Sendra A.; Zamora-Polo F. Exit for Success. Gamifying Science and Technology for University Students Using Escape-Room. A Preliminary Approach. Heliyon 2020, 6 (7), e04340 10.1016/j.heliyon.2020.e04340. 32671257 PMC7341358

[ref5] Alt D. Assessing the Benefits of Gamification in Mathematics for Student Gameful Experience and Gaming Motivation. Comput. Educ. 2023, 200, 104806 10.1016/j.compedu.2023.104806.

[ref6] Stringfield T. W.; Kramer E. F. Benefits of a Game-Based Review Module in Chemistry Courses for Nonmajors. J. Chem. Educ. 2014, 91 (1), 56–58. 10.1021/ed300678f.

[ref7] Ferreiro-González M.; Amores-Arrocha A.; Espada-Bellido E.; Aliaño-Gonzalez M. J.; Vázquez-Espinosa M.; González-de-Peredo A. V.; Sancho-Galán P.; Álvarez-Saura J. Á.; Barbero G. F.; Cejudo-Bastante C. Escape ClassRoom: Can You Solve a Crime Using the Analytical Process?. J. Chem. Educ. 2019, 96 (2), 267–273. 10.1021/acs.jchemed.8b00601.

[ref8] Hanus M. D.; Fox J. Assessing the Effects of Gamification in the Classroom: A Longitudinal Study on Intrinsic Motivation, Social Comparison, Satisfaction, Effort, and Academic Performance. Comput. Educ. 2015, 80, 152–161. 10.1016/j.compedu.2014.08.019.

[ref9] Buckley P.; Doyle E. Gamification and Student Motivation. Interact. Learn. Environ. 2016, 24 (6), 1162–1175. 10.1080/10494820.2014.964263.

[ref10] Dietrich N. Escape Classroom: The Leblanc Process—An Educational “Escape Game.. J. Chem. Educ. 2018, 95 (6), 996–999. 10.1021/acs.jchemed.7b00690.

[ref11] Walsh B.; Spence M. Leveraging Escape Room Popularity to Provide First-Year Students with an Introduction to Engineering Information. Proc. Can. Eng. Educ. Assoc. CEEA 2018, 10.24908/pceea.v0i0.13054.

[ref12] Haimovich I.; Yayon M.; Adler V.; Levy H.; Blonder R.; Rap S. “The Masked Scientist”: Designing a Virtual Chemical Escape Room. J. Chem. Educ. 2022, 99 (10), 3502–3509. 10.1021/acs.jchemed.2c00597.

[ref13] Ang J. W. J.; Ng Y. N. A.; Liew R. S. Physical and Digital Educational Escape Room for Teaching Chemical Bonding. J. Chem. Educ. 2020, 97 (9), 2849–2856. 10.1021/acs.jchemed.0c00612.

[ref14] Peleg R.; Yayon M.; Katchevich D.; Moria-Shipony M.; Blonder R. A Lab-Based Chemical Escape Room: Educational, Mobile, and Fun!. J. Chem. Educ. 2019, 96 (5), 955–960. 10.1021/acs.jchemed.8b00406.

[ref15] Schmidt J.; Amel H.; Korte T.; Beeken M. “In Search of Prof. Aurum”—A Mobile Escape Room Setting for Youth Recreation. J. Chem. Educ. 2023, 100 (8), 3132–3137. 10.1021/acs.jchemed.3c00109.

[ref16] Abdul Rahim A. S. Escape the Desert Island: Blended Escape Rooms in the First-Semester Problem-Based Learning. J. Chem. Educ. 2023, 100 (6), 2459–2465. 10.1021/acs.jchemed.3c00119.

[ref17] Roy B.; Gasca S.; Winum J.-Y. Chem’Sc@pe: An Organic Chemistry Learning Digital Escape Game. J. Chem. Educ. 2023, 100 (3), 1382–1391. 10.1021/acs.jchemed.2c01105.

[ref18] Estudante A.; Dietrich N. Using Augmented Reality to Stimulate Students and Diffuse Escape Game Activities to Larger Audiences. J. Chem. Educ. 2020, 97 (5), 1368–1374. 10.1021/acs.jchemed.9b00933.

[ref19] Elford D.; Lancaster S. J.; Jones G. A. Stereoisomers, Not Stereo Enigmas: A Stereochemistry Escape Activity Incorporating Augmented and Immersive Virtual Reality. J. Chem. Educ. 2021, 98 (5), 1691–1704. 10.1021/acs.jchemed.0c01283.

[ref20] Vergne M. J.; Smith J. D.; Bowen R. S. Escape the (Remote) Classroom: An Online Escape Room for Remote Learning. J. Chem. Educ. 2020, 97 (9), 2845–2848. 10.1021/acs.jchemed.0c00449.

[ref21] de Souza R. T. M. P.; Kasseboehmer A. C. The Thalidomide Mystery: A Digital Escape Room Using Genially and WhatsApp for High School Students. J. Chem. Educ. 2022, 99 (2), 1132–1139. 10.1021/acs.jchemed.1c00955.

[ref22] Cai S. Harry Potter Themed Digital Escape Room for Addressing Misconceptions in Stoichiometry. J. Chem. Educ. 2022, 99 (7), 2747–2753. 10.1021/acs.jchemed.2c00178.

[ref23] Abdul Rahim A. S. Mirror Mirror on the Wall: Escape a Remote Virtual Stereochemistry Lab Together. J. Chem. Educ. 2022, 99 (5), 2160–2167. 10.1021/acs.jchemed.2c00050.

[ref24] Lopez-Pernas S.; Gordillo A.; Barra E.; Quemada J. Comparing Face-to-Face and Remote Educational Escape Rooms for Learning Programming. IEEE Access 2021, 9, 59270–59285. 10.1109/ACCESS.2021.3073601.

[ref25] Heim A. B.; Duke J.; Holt E. A. Design, Discover, and Decipher: Student-Developed Escape Rooms in the Virtual Ecology Classroom. J. Microbiol. Biol. Educ. 2022, 23 (1), e00015-22 10.1128/jmbe.00015-22. 35784618 PMC9249131

[ref26] Barnett J. L.; Cherrette V. L.; Hutcherson C. J.; So M. C. Effects of Solution-Based Fabrication Conditions on Morphology of Lead Halide Perovskite Thin Film Solar Cells. Adv. Mater. Sci. Eng. 2016, 2016, 1–12. 10.1155/2016/4126163.

[ref27] Cherrette V. L.; Hutcherson C. J.; Barnett J. L.; So M. C. Fabrication and Characterization of Perovskite Solar Cells: An Integrated Laboratory Experience. J. Chem. Educ. 2018, 95 (4), 631–635. 10.1021/acs.jchemed.7b00299.

[ref28] Todd C.; Ceballos C. M.; So M. C. Synthesis, Characterization, and Evaluation of Metal-Organic Frameworks for Water Decontamination: An Integrated Experiment. J. Chem. Educ. 2022, 99 (6), 2392–2398. 10.1021/acs.jchemed.2c00115.

[ref29] Yoon S.; Calvo J.; So M. Removal of Acid Orange 7 from Aqueous Solution by Metal-Organic Frameworks. Crystals 2019, 9 (1), 17 10.3390/cryst9010017.

[ref30] Lopez-Pernas S.; Gordillo A.; Barra E.; Quemada J. Escapp: A Web Platform for Conducting Educational Escape Rooms. IEEE Access 2021, 9, 38062–38077. 10.1109/ACCESS.2021.3063711.

