# Supplementary material for: Escaping the Environmental Crises: Online Escape Rooms for Evaluating Student Data Analysis Skills
Source: J Chem Educ. 2023 Oct 23;100(11):4530–5. doi: 10.1021/acs.jchemed.3c00339 (PMC10653218; doi:10.1021/acs.jchemed.3c00339)
Supplement: Supplementary file 1 — ed3c00339_si_001.pdf [file ed3c00339_si_001.pdf]

Supporting Information

Escaping the environmental crises: Online escape rooms for evaluating student data analyses skills

Angelica R. Cash,[a] Julia R. Penick,[a] Celia F. Todd,[b] Monica C. So[a,*]

[a] California State University, Chico, Chico, CA 95929-0210, United States

[b] University of California, Santa Cruz, Santa Cruz, CA 95064, United States

*mso@csuchico.edu

Table of Contents | Pages

Timeline of Virtual Escape Room Activities S2

Troubleshooting Notes to instructor S2

How to Play Virtual Escape Room Activity S3

Renewable Energy Crisis Escape Room Google Form Screenshots & Descriptions S3

Water Decontamination Escape Room Google Form Screenshots & Descriptions S14

Renewable Energy Crisis Pre-lab and Post-lab Confidence Level Questions S15

Escape Room Scenarios with Correct vs. Incorrect Codes S16

Final Report Questions to Evaluate Skills Acquired S18

References S19

Timeline of Virtual Escape Room Activities

Table S1. Proposed timeline of when to implement online escape room assessment during a 16-week academic semester. The virtual escape room activities provided a preview of data and analysis types that students were expected to include in their final group presentations and individual final scientific paper.

Weeks	Group Project
1-6	Renewable Energy Lab Project[S1]
7	Summative Assessment:<br>Renewable Energy Virtual Escape Room
8	Summative Assessment:<br>Group Final Presentation & Individual Final Paper
9-14	Water Decontamination Lab Project[S2]
15	Summative Assessments:<br>Water Decontamination Virtual Escape Room
16	Summative Assessment:<br>Group Final Presentation, Individual Final Paper

Troubleshooting Notes to Instructor

There are a few logistical issues that the instructor should keep in mind to successfully execute these virtual escape rooms. First, there may be times where multiple groups requesting hints at the same time in breakout rooms on a virtual platform like Zoom. One instructor may not be able to address their questions simultaneously or in a timely manner, so to address this, appoint 1-2 experienced student assistants who have completed the virtual escape rooms in prior semesters to assist on days that the virtual escape room assessment is administered. Second, there was not a timer on the Google Forms sheets to track how much time passed during the activity. To overcome this challenge, make sure to release the link to the Google Form escape room at the same time to ensure start times are the same for each breakout group. Google Forms timestamps the submission of the form when students complete the entire assessment. Students may monitor their time by using a timer on their watch or cell phone.

How to Play Virtual Escape Room Activities

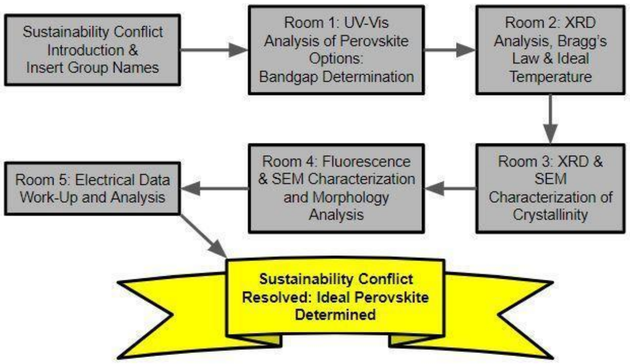

Scheme S1. Flow chart of the escape room process for assessing a student group's data analysis proficiency. Though this is the schematic used in the energy crisis virtual escape room activity, many parts of it are comparable in the water decontamination crisis virtual escape room activity.

Room 1: Spectroscopic Data Analysis

Assessment objective: Evaluate the group's knowledge of interpreting different spectra.
Puzzle: The students analyzed the UV-Vis absorption, fluorescence, and infrared spectra of different nanomaterials used for solving the sustainability crises. For example, they evaluated the absorption pattern of two nanomaterials (e.g. perovskites) to determine if they were adequate semiconductors that would be useful for developing high efficiency solar cells. The students would then be able to solve the energy crisis. After examining the perovskites' absorption patterns in Figure S1, they used the absorption onset wavelength (λ) in Equation S1. Correct calculation of the E_g allowed the students to proceed to Room 2 (Figure S5). Incorrect answers would cause them to remain in the same section.

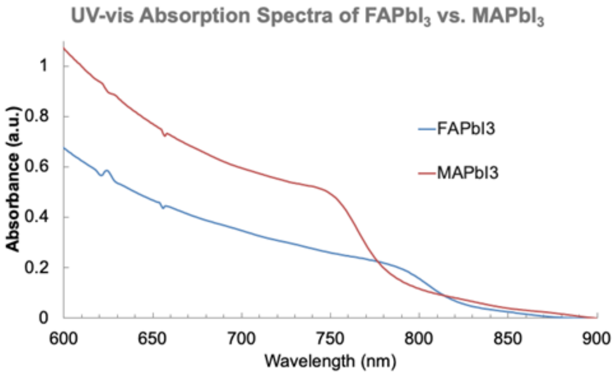

Figure S1. UV-Vis spectra the student groups analyzed in room 1, comparing $FAPbI_3$ and $MAPbI_3$. This is the solid-state absorbance pattern of each perovskite material, from which students derive band gap information from Equation S1.

$$E_g\ (eV) = \frac{1242\ nm \cdot eV}{\lambda\ (nm)}$$

Equation S1. Calculation of the optimal bandgap.

Room 2: Structural Data Analysis

Assessment objective: Assess the group's basic knowledge of evaluating structural data.

Puzzle: The groups were given XRD data to determine structural information about the nanomaterials. For example, to evaluate how far a reaction progressed from starting reagent (lead iodide) to final product (methylammonium lead iodide perovskite) teams had to identify the peaks at 12.5° and 13.9° as originating from lead iodide and perovskite, respectively. Upon evaluating four different XRD spectra (Figure S2), students identified the temperature at which the lead iodide peak is no longer present. Finally, the students used Bragg's law (Equation S2) to predict where the third order peak (n = 3) would appear in degrees, based on the wavelength (λ), interplanar spacing of crystals (d), and angle at which diffraction peaks occur (θ). Upon all correct answers, the group would proceed to Room 3 (pp. S8).

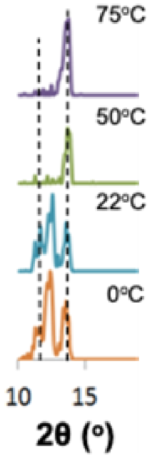

Figure S2. XRD that was to be analyzed by students in Room 2.

$$n\lambda = 2d\sin\theta$$

Equation S2. Bragg's law.

Room 3: Morphological Data Analysis

Assessment objective: Assess the students' abilities to analyze morphological data.

Puzzle: Students used SEM images to conduct a morphological data analysis of the nanomaterials. For instance, the students were provided with SEM data in which two different anti-solvents were used to promote crystallization of perovskites (Figure S3). Students need to

choose the anti-solvent that produces higher crystalline materials. Input of all correct answers would allow the groups to proceed to Room 4 (pp. S9-S12).

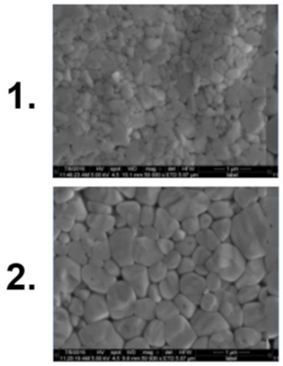

Figure S3. SEM provided for students to analyze crystallinity in Room 3.

Room 4: Elemental Composition Data Analysis

Assessment objective: Assess the group's ability to interpret elemental composition data.
Puzzle: During Lab 2, students were provided EDXS data for four different nanomaterials. Students had to identify one of the four unknown nanomaterials by examining the elements present in Table S2. They also needed to rationalize why there were extra elements that did not belong in the composition of the nanomaterial. Once they chose the correct answers, the group would proceed to Room 5 (pp. S13).

Element	Mass %
C	64.92
N	31.37
O	2.01
Co	1.69

Table S2. EDXS Results

Room 5: Performance Data Analysis

Assessment objective: Assess the groups' abilities to evaluate the performance data of the given nanomaterials.
Puzzle: Students were provided with electrical performance data (Lab 1). To demonstrate their understanding of this data analysis, students utilized Equation S3 to calculate the average efficiency of the sample cells given. Upon correct input of the average efficiency, the group proceeded forward and resolved the energy crisis.

$$\eta = \frac{V_{OC} I_{SC} FF}{P_{in}} \quad x100\%$$

Equation S3. Equation for calculating electrical efficiency (η): V_{oc} is the open circuit voltage. I_{sc} is the short circuit current, FF is the fill factor and P is the input power.

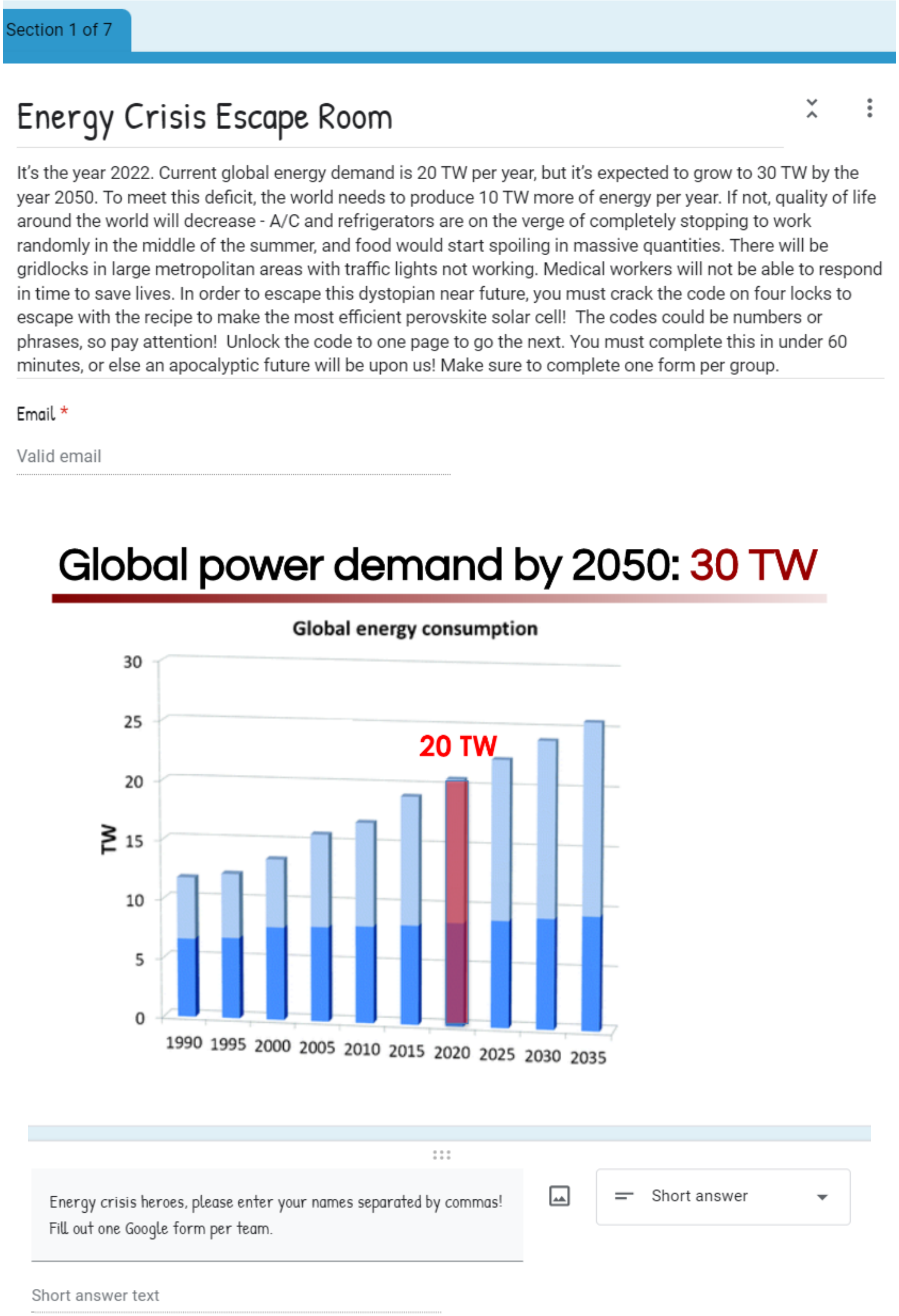

Section 1 of 7

Energy Crisis Escape Room

It's the year 2022. Current global energy demand is 20 TW per year, but it's expected to grow to 30 TW by the year 2050. To meet this deficit, the world needs to produce 10 TW more of energy per year. If not, quality of life around the world will decrease - A/C and refrigerators are on the verge of completely stopping to work randomly in the middle of the summer, and food would start spoiling in massive quantities. There will be gridlocks in large metropolitan areas with traffic lights not working. Medical workers will not be able to respond in time to save lives. In order to escape this dystopian near future, you must crack the code on four locks to escape with the recipe to make the most efficient perovskite solar cell! The codes could be numbers or phrases, so pay attention! Unlock the code to one page to go the next. You must complete this in under 60 minutes, or else an apocalyptic future will be upon us! Make sure to complete one form per group.

Email *

Valid email

Energy crisis heroes, please enter your names separated by commas! Fill out one Google form per team.

Short answer

Short answer text

Figure S4. Renewable Energy Crisis Escape Room Google Form screenshot.

Section 2 of 7

UV-Vis Absorption Characterization

You're under a time crunch and need to unlock information about your perovskite-based solar cells. To start, you collect some UV-Vis data for your best two perovskites--formamidinium lead iodide (FAPbI3) and methylammonium lead iodide (MAPbI3)--and plot their data to compare the two. You must analyze this data to carry forth the best perovskite solar cell to the next step. The resulting plot is as follows:

UV-Vis Plot

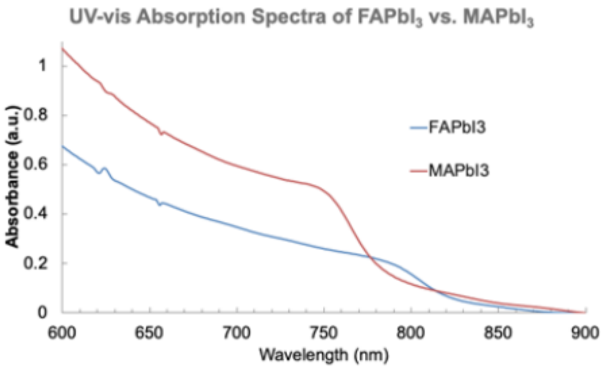

What is the optical band gap for FAPbI3 (in eV)? Include number only and not units. *

Short answer text

What is the optical band gap for MAPbI3 (in eV)? Include number only and not units. *

Short answer text

Based on the band gaps calculated, which of the two would be a better semiconductor, FAPbI3 or MAPbI3? *

- ◯ FAPbI3
- ◯ MAPbI3

Figure S5. Room 1: UV-Vis Data Characterization

Section 3 of 7

XRD Characterization

Great! You unlocked the UV-vis characterization data! Your next mission is to characterize the XRD data for your best perovskite samples while comparing the varying temperatures. Then, you narrow in on the following results of your XRD scan:

XRD of sample:

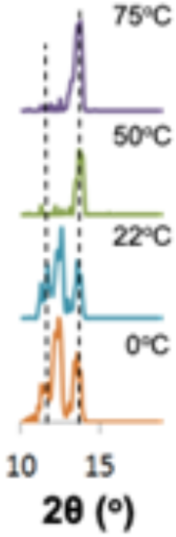

What is the peak at 12.5°? (Two word answer; make sure to type lower-case letters.)

Short answer text: ___________________

What is the peak at 13.9°? (One word answer; make sure to type lower-case letters.)

Short answer text: ___________________

At what temperature does the 12.5° peak disappear? Include numbers only and not units.

Short answer text:______________________

Predict at what 2θ value the 3rd order reflection would appear based on Bragg's Law. Assume the wavelength of 1.54 Angstroms. Include numbers only and not units.

Short answer text: ______________________

Section 4 of 7

You're on a roll! You have the ideal annealing temperature, now carry this forward to determine the best anti-solvent for crystallization. Here, dimethylformamide (DMF) and 2-methoxyethanol (2-ME) were applied as anti-solvents to see how they affect the perovskite's crystallinity. Your mission is to combine the XRD and SEM data sets to figure out what's going on!

DMF & 2ME XRD

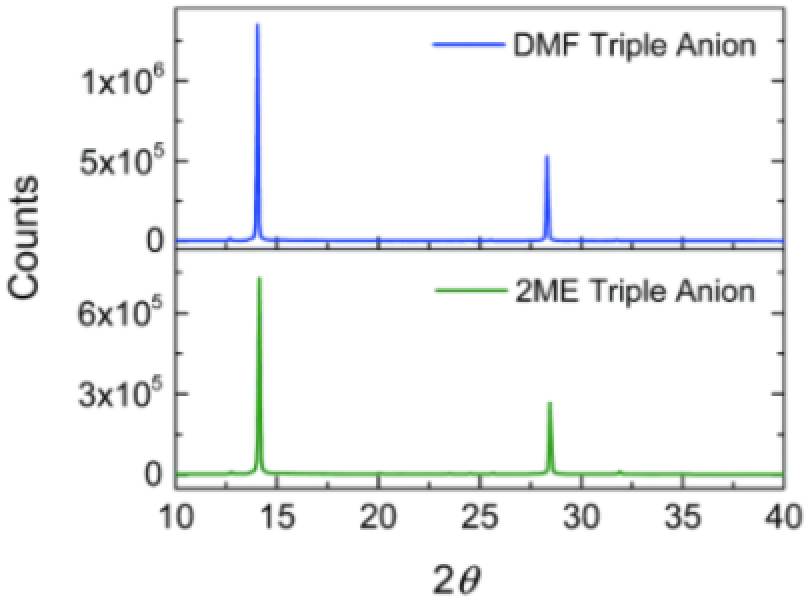

DMF & 2ME SEM

1

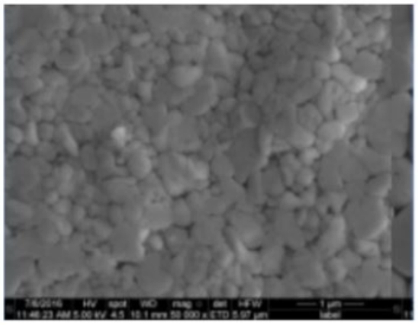

2

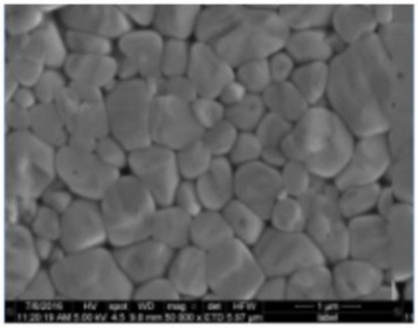

Which SEM image matches the DMF triple anion XRD pattern? Use numbers corresponding to the SEM image.

Short Answer Text: _______________

Which SEM image matches the 2-ME triple anion XRD pattern? Use numbers corresponding to the SEM image.

Short Answer Text: _______________

Which antisolvent-treated perovskite sample has higher crystallinity (2-ME or DMF)? Use anti-solvent abbreviations in caps; exclude "TA" from your answer.

Short Answer Text: _______________

Which of these perovskite samples would you prefer for actual use? Use numbers corresponding to the SEM image.

Short Answer Text: _______________

Section 5 of 7

Fluorescence & SEM Characterization

Amazing! Your next mission is to check the quality and morphology of the perovskite via fluorescence (PL) and SEM analyses. You first obtain the following PL plots, followed by the following SEM images.

PL Plot

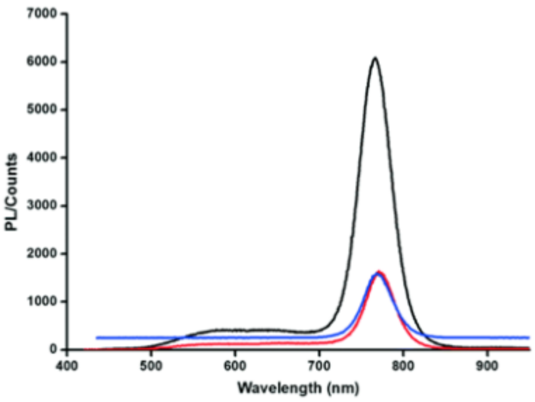

Where is your maximum emission wavelength (in nm) for the black plot and the blue plot? You only need to enter the number, not units.

Short Answer Text: ________________

Where is the expected excitation wavelength (in nm)? You only need to enter the number, not units.

Short Answer Text: ________________

SEM Sample A

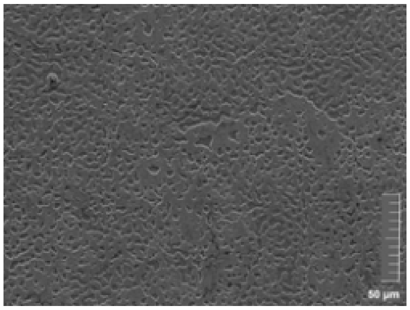

Which color PL plot does this SEM image belong with? (One word answer; make sure to type lower-case letters.)

Short Answer Text: ________________

SEM Sample B

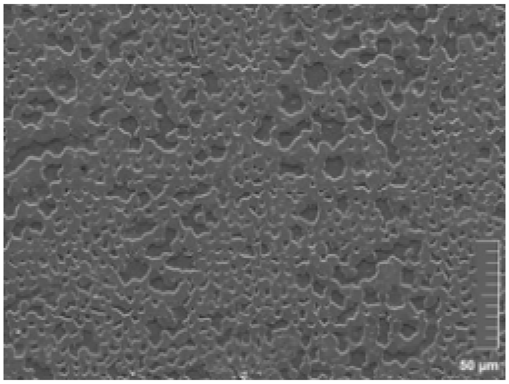

Which color PL Plot does this SEM image belong with? (One word answer; make sure to type lower-case letters.)

Short Answer Text:________________

Which of these perovskite samples would you prefer for actual use - Sample A or Sample B?

Short Answer Text: ________________

Which of the following scenarios would apply to the blue and red plots?

Multiple Choice:

A)The two were synthesized using the ratio of PbI2 to MAI.

B)The two were synthesized with similar annealing times.

C)The two were synthesized using the same anti-solvent

D)The two were synthesized at the same temperature.

E)All of the above.

Which of the following scenarios would apply to the black and red plots?

Multiple Choice:

A)One was annealed at 70°C and another at 145°C.

B)One was treated with chlorobenzene anti-solvent, while another was not.

C)One was annealed for 20 mins, while the other was annealed for 40 minutes.

D)All of the above.

Section 6 of 7

Electrical Characterization

You see the light at the end of the tunnel! Finally, you are ready to unlock the capabilities of your new perovskite solar cell! You begin collecting the data and obtain the following results:

Electrical Data & Dimensions

Sample	Voltage (V)	Resistance (kilo-ohms)	Average Area (cm^2)
1	0.033	0.820	0.0305
2	0.157	2.533	0.0314
3	0.061	2.590	0.0359
4	0.053	7.033	0.0278

Given the following electrical information and assuming the fill factor is 0.75, calculate the average efficiencies (in percentage form) of all 4 samples. Exclude the % symbol. Please input ONE value.

Short answer: ___________________

Section 7 of 7

Congratulations!

You have escaped a dystopian future with the recipe to make perovskite solar cells and save mankind from a global energy deficit! You are an energy crisis hero! Please notify your instructor of the good news!

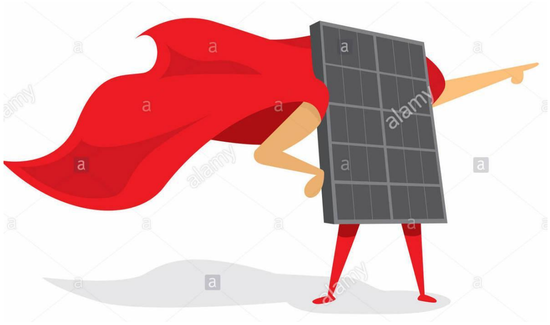

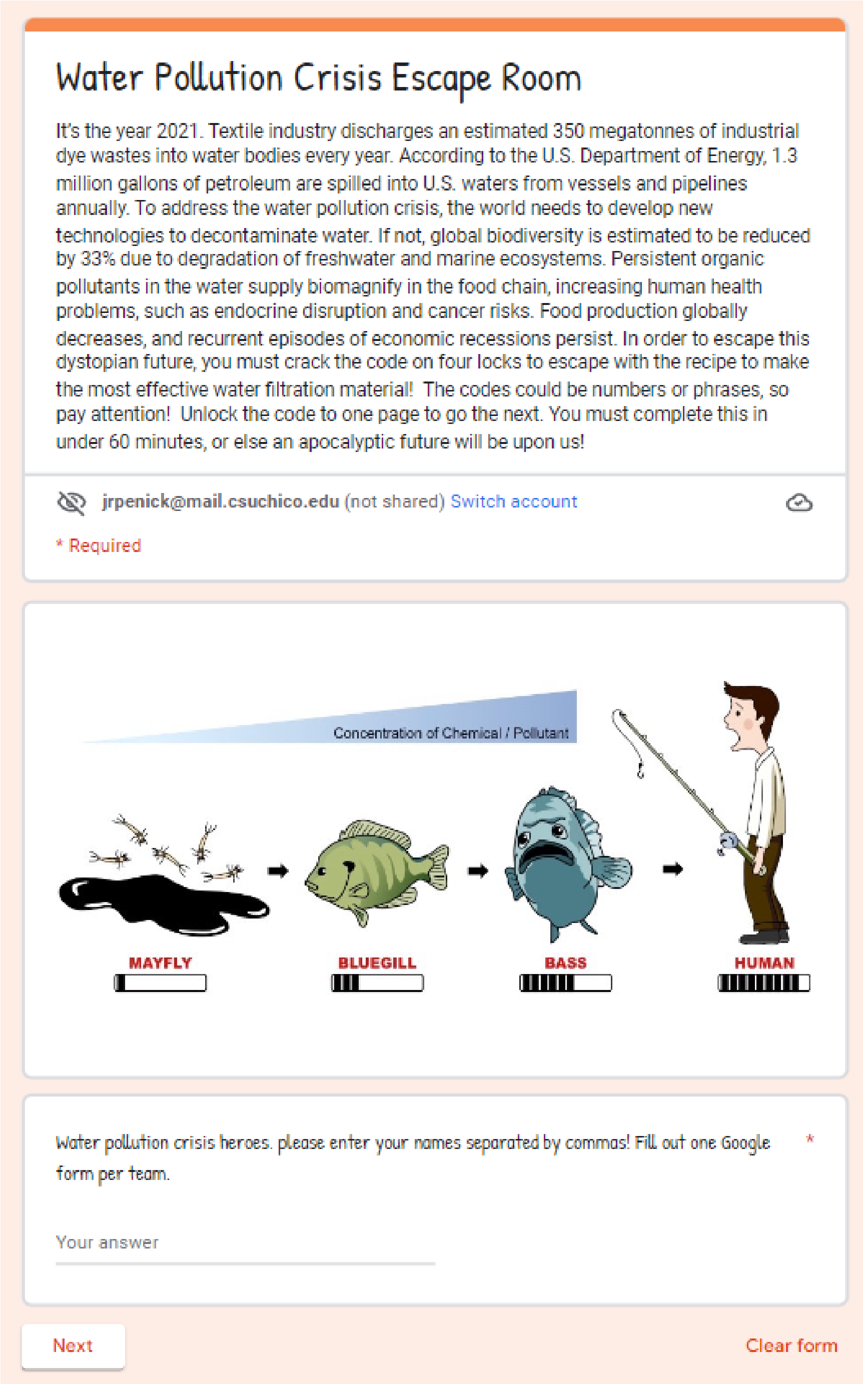

Water Pollution Crisis Escape Room

It's the year 2021. Textile industry discharges an estimated 350 megatonnes of industrial dye wastes into water bodies every year. According to the U.S. Department of Energy, 1.3 million gallons of petroleum are spilled into U.S. waters from vessels and pipelines annually. To address the water pollution crisis, the world needs to develop new technologies to decontaminate water. If not, global biodiversity is estimated to be reduced by 33% due to degradation of freshwater and marine ecosystems. Persistent organic pollutants in the water supply biomagnify in the food chain, increasing human health problems, such as endocrine disruption and cancer risks. Food production globally decreases, and recurrent episodes of economic recessions persist. In order to escape this dystopian future, you must crack the code on four locks to escape with the recipe to make the most effective water filtration material! The codes could be numbers or phrases, so pay attention! Unlock the code to one page to go the next. You must complete this in under 60 minutes, or else an apocalyptic future will be upon us!

jrpenick@mail.csuchico.edu (not shared) Switch account

* Required

Water pollution crisis heroes, please enter your names separated by commas! Fill out one Google form per team. *

Your answer

Next

Clear form

Figure S6. Water Decontamination Crisis Escape Room Google Form screenshot.

How confident are you with your knowledge of the following laboratory techniques?

	Very confident	Confident	Somewhat confident	Slightly confident	Not very confident
Cleaning substrates	○	○	○	○	○
spincoating	○	○	○	○	○
annealing	○	○	○	○	○
solid-state UV-vis spectroscopy	○	○	○	○	○
solid-state fluorescence spectroscopy	○	○	○	○	○
X-ray diffraction	○	○	○	○	○
current density-voltage mapping	○	○	○	○	○

Figure S7. Sample pre- and post-activity confidence level questions.

Renewable Energy Crisis Escape Room Screenshots (Correct vs. Incorrect Codes)

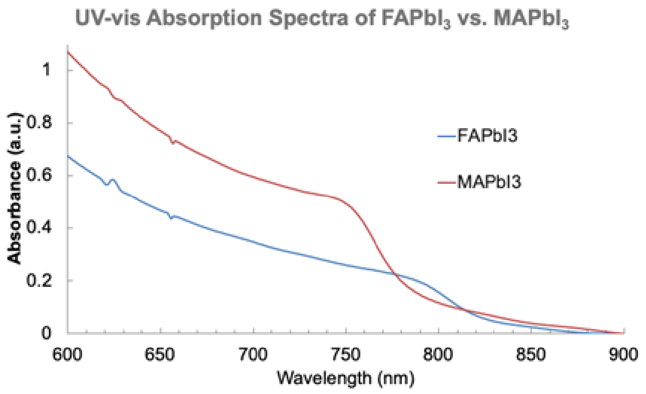

What is the optical band gap for FAPbI3 (in eV)? Include number only and not units. *

1.48

What is the optical band gap for MAPbI3 (in eV)? Include number only and not units. *

1.55

Based on the band gaps calculated, which of the two would be a better semiconductor, FAPbI3 or MAPbI3? *

- (selected) FAPbI3
- MAPbI3

XRD Characterization

Great! You unlocked the UV-vis characterization data! Your next mission is to characterize the XRD data for your best perovskite samples while comparing the varying temperatures. Then, you narrow in on the following results of your XRD scan:

Figure S8. Correct escape room “codes” allow users to move onto the next “room”, where a congratulatory message provides a new task or “mission” to complete.

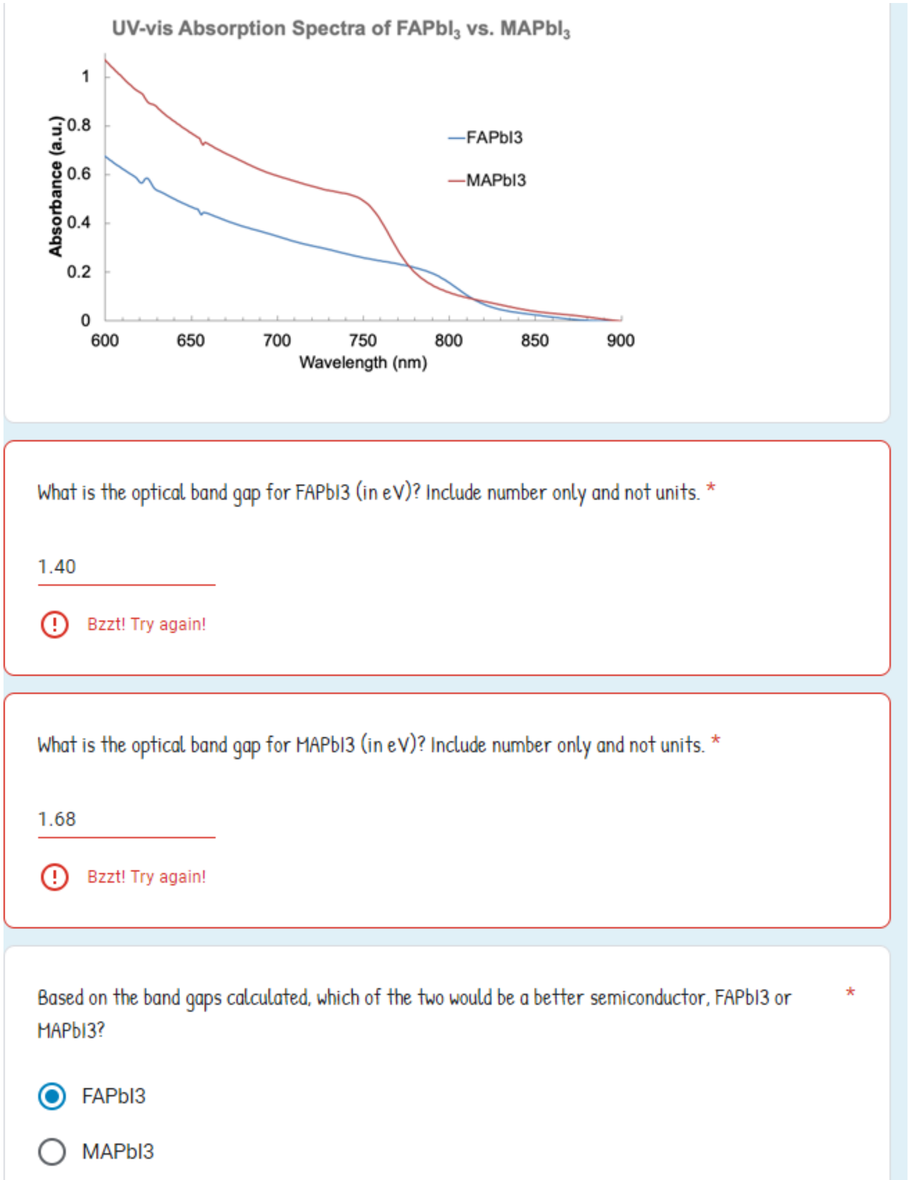

Figure S9. Incorrect escape room “codes” yield error messages, preventing users from proceeding to the next page, or “room” of the escape room.

Questions Incorporated into Final Report (Renewable Energy Lab[S1]):

1. Discuss the synthesis and structure of perovskites, focusing on stoichiometry.
2. Why is the perovskite layer sandwiched between the TiO2 and CuSCN layers? What would happen if the TiO2 was sandwiched between the perovskite and CuSCN layers? Also, explain whether we used FTO as both the cathode and anode?
3. Can the spectroscopic or electrical data obtained for your samples provide insight into which layer absorbs light? Explain.
4. Why do some solar cells work and others do not? Support with experimental data. In terms of the fabrication process, discuss one modification each to (a) minimize short-circuiting and (b) improve sample reproducibility.
5. Calculate the power conversion efficiency of your solar cells. Assume available solar intensity to be 100 mW/cm2. Measure active surface area of the cell with a ruler.
6. Tabulate the output (current, voltage) of three cells. Explain the losses incurred when connecting solar cells in series.
7. Discuss at least two potential modifications to improve the photovoltaic efficiency.
8. Should we use lead in perovskite-based solar cells in the future? If so, explain why. If not, discuss alternatives.

Questions Incorporated into Final Report (Water Decontamination Lab[S2]):

1. Discuss the syntheses and structure of the four MOFs.
2. Why is the hydrochloric acid used for synthesis of the UiO MOFs but not the ZIF MOFs? What would happen if base were added to the reaction of UiO MOFs instead? Also, predict what would happen if we used 0.1 M instead of 6 M hydrochloric acid.
3. Can the spectroscopic, diffraction, or microscopy data obtained for your samples provide insight into which MOFs ad/absorb dye or oil? Explain.
4. Why do some MOFs ad/absorb more dyes or oil than others? Support with experimental data. In terms of the reaction, discuss one modification each to improve (a) crystallinity, (b) porosity, and (c) water stability.
5. What are the possible ad/absorption mechanisms of POPs in the MOFs? Explain.
6. Which of the four MOFs had the highest ad/absorption capacity for POPs? Explain.
7. Discuss at least two potential modifications to improve the ad/absorption of POPs to the MOFs.
8. What other metals can be used to form the ZIF MOFs? What other linkers can be used to form the UiO MOFs?

References

S1. Cherrette, V.L., Hutcherson, C.J., Barnett, J.L. and So, M.C. Fabrication and characterization of perovskite solar cells: An integrated laboratory experience. *J. Chem. Educ.*, **2018,** *95(4)*, 631-635.

S2. Todd, C., Ceballos, C.M. and So, M.C. Synthesis, Characterization, and Evaluation of Metal–Organic Frameworks for Water Decontamination: An Integrated Experiment. *J. Chem. Educ.*, **2022**, *99(6)*, 2392-2398.
